# miR-424 inhibits apoptosis and inflammatory responses induced by sevoflurane through TLR4/MyD88/NF-κB pathway

**DOI:** 10.1186/s12871-022-01590-z

**Published:** 2022-02-23

**Authors:** Zeyu Li, Tao Wang, Yonghao Yu

**Affiliations:** 1 Department of Anesthesiology, Tianjin Baodi Hospital, Baodi Clinical College of Tianjin Medical University, Tianjin, 301800 China; 2 Graduate College, Tianjin Medical University, Tianjin, 301800 China; 3 Department of Anesthesiology, Tianjin Chest Hospital, Tianjin, 300222 China; 4 Department of Anesthesiology, Tianjin Medical University General Hospital, 154 Anshan Road, Heping District, Tianjin, 362255 China

**Keywords:** Sevoflurane, miR-424, Apoptosis, Inflammatory, TLR4

## Abstract

**Background:**

Side effects of sevoflurane in anterograde and retrograde memory have been widely reported. However, there is no convincing evidence that sevoflurane directly causes the development of neurotoxicity. miR-424 has the potential to regulate the neurotoxicity caused by isoflurane anesthesia, and it has a complementary sequence with the 3’UTR region of TLR4. Thus, our study aims to explore whether sevoflurane directly causes neurotoxicity, the effects of miR-424 on sevoflurane induced apoptosis and inflammation, and the underlying mechanism.

**Methods:**

Sevoflurane effects were identified both in mouse and in PC12 cells. Western blots and ELISA were used for protein detection, while micro (mi) RNA expression was measured with RT-qPCR. Dual luciferase reporter assays were employed to study the interaction between miR-424 and Toll-like receptor 4 (TLR4) using miR-424 mimics and TLR4 over-expression.

**Results:**

Sevoflurane stimulated expression of Bax2 and Caspase-3, and increased apoptosis ratio both in vivo and vitro (*P* < 0.05). Inflammatory cytokines, such as tumor necrosis factor (TNF)-α, interleukin (IL)-1β and IL-6, were up-regulated by sevoflurane, while IL-10 was downregulated (*P* < 0.05). Sevoflurane treatment enhanced the phosphorylation of NF-κB, and up-regulated the expressions of TLR4 and MyD88 (*P* < 0.05), which demonstrated that sevoflurane inhibited proliferation and differentiation of neuronal cells by activating TLR4/MyD88/NF-κB signaling both in vitro and vivo. However, up-regulation of miR-424 attenuated the negative effects of sevoflurane by targeting the 3′-untranslated region (UTR) of TLR4 and inducing the degradation of mRNA (*P* < 0.05).

**Conclusions:**

In vitro, sevoflurane induces activation of the endogenous TLR4 signaling pathway, thereby promoting apoptosis and inflammatory cytokine expression. Exogenous TLR4 acts as an agonist to stimulate TLR4 signaling, whereas miR-424 inhibits both endogenous and exogenous TLR4 signaling, thereby preserving proliferation and differentiation of neuronal cells.

## Introduction

Sevoflurane is a common inhaled anesthetic agent used during intravenous anesthesia, especially in pediatric surgery because of its high induction efficiency and quick recovery. Hypnosis, amnesia, analgesia, dyspraxia and autonomic block can be provided by sevoflurane during surgical and procedural interventions [1]. The precise mechanism of sevoflurane in inducing and maintaining general anesthesia is unknown. Sevoflurane transfers from inspired gas via the pulmonary capillaries into the blood, thereby circulating to the brain. Sevoflurane dilates cerebrovascular vessels, thereby increasing intracranial pressure and reducing metabolic rate [1, 2]. Sevoflurane has potential neurotoxicity, nephrotoxicity and hepatotoxicity; however, there is no convincing evidence that sevoflurane directly causes the development of neurotoxicity [2].

Apoptosis is a critical mode of cellular programmed death. Bcl-2 family members Bax and Bak regulate the internal apoptotic pathway of cells and induce them to enter the apoptotic process [3, 4]. Caspases play crucial roles in mediating programmed cell death (apoptosis), of which caspase-3 is the most frequently activated protease that works by catalyzing cleavage of key cellular elements. Caspase-3 is critical for brain development, chromatin aggregation and DNA fragmentation during the process of apoptosis [5].

Human interleukin (IL)-1α, IL-1β, IL-6 and tumor necrosis factor-α (TNF-α) are proinflammatory cytokines known to induce tissue factor expression and activity [6], while IL-4, IL-10 and IL-13 are known as inhibitors of IL-1α/β both at the mRNA and protein levels [7, 8]. Toll-like receptors (TLRs) have a leucine-rich extracellular repeat region and a cytoplasmic tail containing a Toll-like receptor (TIR) domain. TLRs identified on different microbial surfaces or on intracellular components enable an organism to develop acquired immunity by stimulating innate immunity. The TLR signaling pathway originates from the TIR region. Myeloid differentiation factor 88 (MyD88), TIR domain containing adaptor protein (TIRAP) and TIR-domain-containing adapter-inducing interferon-β (TRIF) act as adapters for the TLR conserved domain to regulate the TLR signaling pathway [9–11].

TLR2 and TLR4 regulate self-renewal and the fate of neuronal progenitor cells, mediated by MyD88 and nuclear factor kappa-B (NF-κB) pathways. TLR4, the first mammalian TLR homologous protein, inhibits proliferation and differentiation of neuronal cells. In contrast, TLR2 deficiency impairs hippocampal neurogenesis [11–14]. TLR plays its specific role in neuronal progenitor cells through the NF-κB signaling pathway [10, 14].

TLRs signaling pathways are widely classified as MyD88-dependent and -independent. MyD88 is an adaptor recruited by TLRs with the exception of TLR3 [10, 12, 13]. Activation of NF-κB and AP-1 transcription factors leads to the expression of IL-6, IL-1 and TNF-α [7]. TLR4 triggers downstream signaling by kinases which activate AP-1 and NF-κB. MyD88 mediates the production of cytokines and is therefore critical to the TLR signaling pathway [10–12].

Micro (mi) RNAs are small non-coding RNAs that have tumor-suppressive and carcinogenic properties. Mature miRNAs lead to degradation or translational inhibition of targeted mRNAs by binding to specific bases. miRNAs are involved in the regulation of multiple molecular pathways, and play important roles in cell growth, differentiation and apoptosis. miR-424 plays a regulatory role in a variety of tumors or diseases. It not only inhibits the invasion and migration of intrahepatic cholangiocarcinoma cells [15], but also increases the sensitivity of gastric cancer cells to cisplatin [16]. Bioinformatics study has found that miR-424 is one of the differentially expressed weighted genes in isoflurane-induced general anesthesia, suggesting that it may be involved in the neurotoxicity caused by the isoflurane anesthesia [17]. Therefore, we reasonably speculate that miR-424 may be related to the occurrence and development of sevoflurane induced neurotoxicity.

## Materials and methods

### Antibody

The following materials were used: anti-Bcl-2 antibody (Abcam, 182,858), anti-Bax antibody (Abcam, 32,503), anti-GAPDH antibody (Abcam, 8245), anti-cleaved caspase-3 antibody (Cell Signaling Technology, 9664), anti-IL-1β antibody (Cell Signaling Technology,12,242), anti-IL-6 antibody (Cell Signaling Technology, 12,912), anti-IL-10 antibody (Abcam, 189,392), anti-TNF-α antibody (Abcam, 183,218), anti-TLR4 antibody (Abcam, 22,048), anti-MyD88 antibody (Abcam, 133,739), anti-NF-κB antibody (Abcam, 16,502), anti-p-NF-κB antibody (Abcam, 76,302), goat anti-rabbit HRP-conjugated antibody (Abcam 6721), and goat anti-mouse HRP-conjugated antibody (Abcam 6789).

### Animals and treatments

Animal experiments were followed the guidelines of the National Institute of Health Guide for the Care and Use of Laboratory Animals [18], and all animal experiments were performed in the Laboratory Animal Center of Tianjin Baodi Hospital. A total of 24 adult male Sprague-Dawley rats (from Tianjin Baodi Hospital) were divided into four groups (*n* = 6): control (con), sevoflurane (Sev), sevoflurane combined with negative control oligonucleotide (NC), and sevoflurane combined with miR-424 mimics (Sev + mimic) [19]. For sevoflurane treatment, 2% sevoflurane (Maruishi Pharmaceutical Co., Ltd.) was administered by inhalation for 15 min. miR-424 mimics and control oligonucleotides were first modified and conjugated to cholesterol, then rats were treated with miR-424 mimics (1 nm in 5 μl) or control oligonucleotides (4 nm in 5 μl) injected into the dorsal hippocampus. The rats were euthanatized 72 h later and hippocampal tissues were obtained [19, 20]. All miR-424 mimics, NC oligonucleotides and modified miRNA agents were obtained from RiboBio Company in Guangzhou, China.

### TUNEL assay

Rats were anesthetized with intraperitoneal injections of 40 mg/kg sodium pentobarbital before euthanasia. Hippocampal tissues were dissected and fixed with 4% paraformaldehyde overnight at room temperature (RT), then were paraffin-embedded, dehydrated and cut into 4-μm sections. After deparaffinization with xylene and rehydration, a terminal deoxynucleotidyl transferase dUTP nick end labeling (TUNEL) kit (OriGene Technologies, Inc.) was utilized for quantification of apoptosis in five randomly chosen visual areas by microscopic examination (BX50/Olympus Corporation). Apoptosis-positive cells were stained brown-yellow, while normal nuclei were stained blue [19].

### RT-PCR

RNA isolation was performed using the miRNeasy Mini kit (Qiagen, Germany). Reverse transcription was carried out with the TaqMan MicroRNA Reverse Transcription kit (Thermo Fisher Scientific, Inc.). hsa-miR-424 primers were applied to the CFX96 Touch™ Real-Time PCR Detection System (Bio-Rad, USA). Primer sequences were as follows: U6 forward primer (FP), 5′-GTGCTCGCTTCGGCAGCACATATAC-3′, reverse primer (RP), 5′-AAAAATATGGAACGCTTCACGAATTTG-3; GAPDH FP, 5′-ACCCACTCCTCCACCTTTGA-3′, RP, 5′-CTGTTGCTGTAGCCAAATTCGT-3′; hsa-miR-424 FP, 5′-CGCAAAACGTGAGGCGCT-3′ -3′, RP, 5-CCAGTGCAGGGTCCGAGGTA-3′; TLR4 FP, 5′-AATGGATCAAGGACCAGAGG-3′, RP, 5′-CAGCCAGCAAGAAGCATCAG-3′. The PCR program consisted of 30 s at 95 °C, followed by 45 cycles of 5 s at 95 °C and 58 °C for 34 s. The expression level of miRNA was obtained using the 2 − ∆∆Cq method with U6 small nuclear RNA as control, and GAPDH as internal control for TLR4 [21].

### Cell culture

PC12 cells and neuronal medium were obtained from ScienCell Research Laboratories (USA). Cells were cultured at 37 °C in 95% humidity and 5% CO2. 6 different treatments which cell underwent are as follows: sevoflurane (Sev), negative control oligonucleotide (NC), miR-424 mimic (mimic), pLenti-GIII-CMV vector (pcDNA), and pLenti-GIII-TLR4 plasmid (TLR4) [21].

### Transfection

Transfections were performed with 5 nM hsa-miR-424 mimics (5′-UAAGUGCUUCCAUGCUU-3′), or negative control oligonucleotides (5′-UCACAACCUCCUAGAAAGAGUAGA-3′) in density of 5 × 104 cells per cm2 using Lipofectamine® 3000 according to the manufacturer’s instructions. All these oligoes and reagent above were from Thermo Fisher Scientific (USA). For TLR4 overexpression, the pLenti-GIII-CMV vector was obtained from Applied Biological Materials (Canada). RT-qPCR was performed 72 h after transfection to determine transfection efficiency [21].

### Exposure to sevoflurane

Culture plates were incubated in an airtight chamber with inlet and outlet pipes. The inlet was connected to a sevoflurane vaporizer. The chamber was gassed with 0% (control group) or 8% (sevoflurane group) sevoflurane in air (95% air/5% CO2) for 15 min. The concentration of sevoflurane was controlled with a monitor (Drager, Germany). After sealing, the chambers were incubated at 37 °C for 6 h. Gas in the chamber was replenished every 3 h [19].

### Apoptotic cell analysis

Cell apoptosis was determined by flow cytometry. After harvesting, cells were washed twice with PBS and then assayed with the Annexin V-FITC Apoptosis Detection Kit (Becton–Dickinson, USA). Briefly, after being resuspended in 1× binding buffer, cells were stained with propidium iodide and annexin V. The apoptosis ratio was measured with the BD FACS Accuri C6 (Becton-Dickinson) [21].

### Western blot assay

After collection, cells were washed twice with cold PBS, lysed for 15 min on ice with lysis buffer (Beyotime, China) containing cocktail (proteinase inhibitor) (Merck, Germany). Brain tissues were sonicated and supernatants were reserved after centrifugation at 13000×g for 30 min. Protein concentration was assayed with a kit (Beyotime). From each sample, 1 g protein was resolved by SDS-PAGE and transferred to PDF membranes (Whatman, Germany). After blocking in PBS containing 5% milk at RT for 60 min, the membranes were incubated in primary antibody solution overnight at 4 °C. After washing twice for 5 min per wash with TBST, the membranes were incubated with secondary antibodies at RT for 1 h. Protein bands were visualized with an ECL detection kit (PerkinElmer, USA), and filmed in a C-DiGit Blot Scanner (Li-cor Bioscience, USA). Protein staining was quantified with Image Studio Digits v. 3.1 [20].

### Luciferase assays

The 3′-untranslated regions (UTR) of TLR4 were cloned into the Renilla luciferase plasmid pRL-TK vector (Promega, USA). Mutated 3′-UTR was produced with the QuikChange II Site-Directed Mutagenesis kit (Catalog: #200524, Agilent Technologies, USA). For luciferase assays, 105 HN-h (Human hippocampal nerve) cells per well were seeded, and miR-424 mimics (5 nM), pRL-TK vector (10 ng) and luciferase plasmid pRL-TK vector (10 ng) were co-transfected with Lipofectamine® 3000. After 48 h, the cells were harvested and lysed with the Dual-Luciferase Reporter Assay System (Promega, USA) followed by measurement of luciferase activity. Renilla luciferase was used for normalization [21].

### Statistical analysis

Each experiment was repeated independently at least three times. Differences among three or more groups were analyzed with one-way analysis of variance (ANOVA). If the results indicated a significant difference, analysis was then continued with a post hoc multiple comparisons Bonferroni′s test with Prism 6 (GraphPad Software, USA). A *P* < 0.05 was considered statistically significant [20].

## Results

### miR-424 rescues apoptosis induced by sevoflurane in vivo

**Fig. 1:**
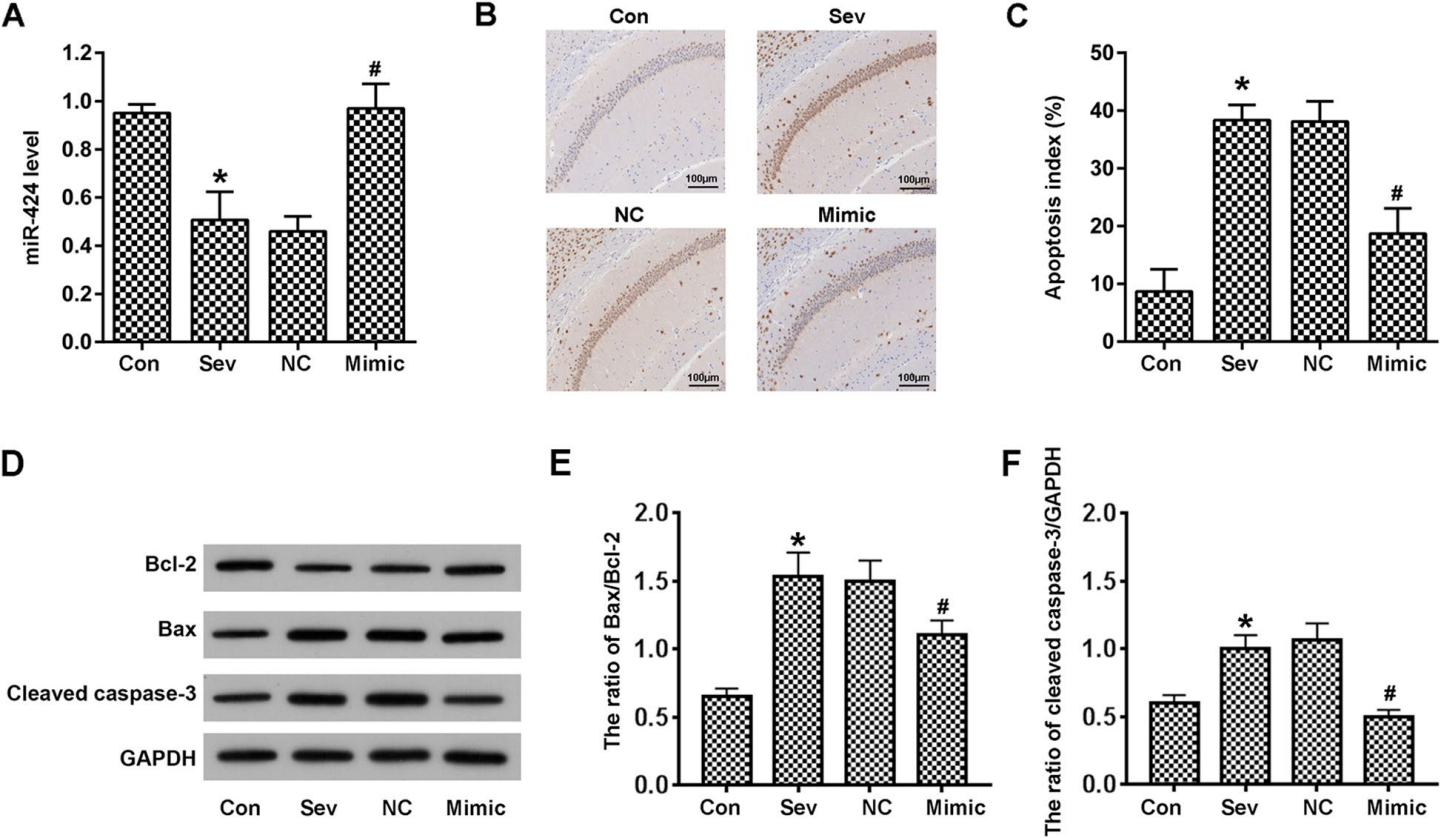
miR-42 inhibits hippocampal apoptosis in sevoflurane-treated rats. **A**. Detection of miR-424 expression in vivo with RT-PCR. **B**-**C**. Apoptosis detection by TUNEL assay. The percentage of apoptotic cells was calculated by cell counts. **D**-**F**. Western blot was used to detect apoptosis-associated proteins. Images were quantified with image J software, and the ratios of Bax/Bcl-2 and cleaved caspase-3/GAPDH were calculated. *n* = 8; *p < 0.05 vs control; ^#^*p* < 0.05 vs NC

We examined the mechanism of sevoflurane action in the rat hippocampus using different experimental groups. The control group was treated with normal saline, while the other groups were treated with sevoflurane. The NC group was additionally treated with a commercial miRNA sequence to remove background, while the Mimic group was treated with an miR-424 mimic sequence. After treatment, the hippocampi from the different groups were collected for RNA and protein extraction, paraffin embedding and sectioning. RT-PCR was performed to detect miR-424 expression. Compared with the control group, miR-424 expression was decreased by 50% after sevoflurane treatment, while this effect was reversed by exogenous addition of mimic miR-424 (Fig. 1A). TUNEL staining was used to detect apoptosis, which was significantly increased after sevoflurane, while this trend was reversed by miR-424 addition (Fig. 1B and C).

For further verification of this effect of sevoflurane, western blots were prepared for the detection of apoptosis-related factors. Compared with the control group, sevoflurane significantly decreased the protein level of Bcl-2. In contrast, apoptotic factors including Bax and Caspase-3 were up-regulated, which indicated that cells had been induced into an apoptotic progression. It should be noted that these trends were reversed by miR-424 treatment (Fig. 1D-F).

### miR-424 inhibits inflammatory responses induced by sevoflurane in vivo

**Fig. 2:**
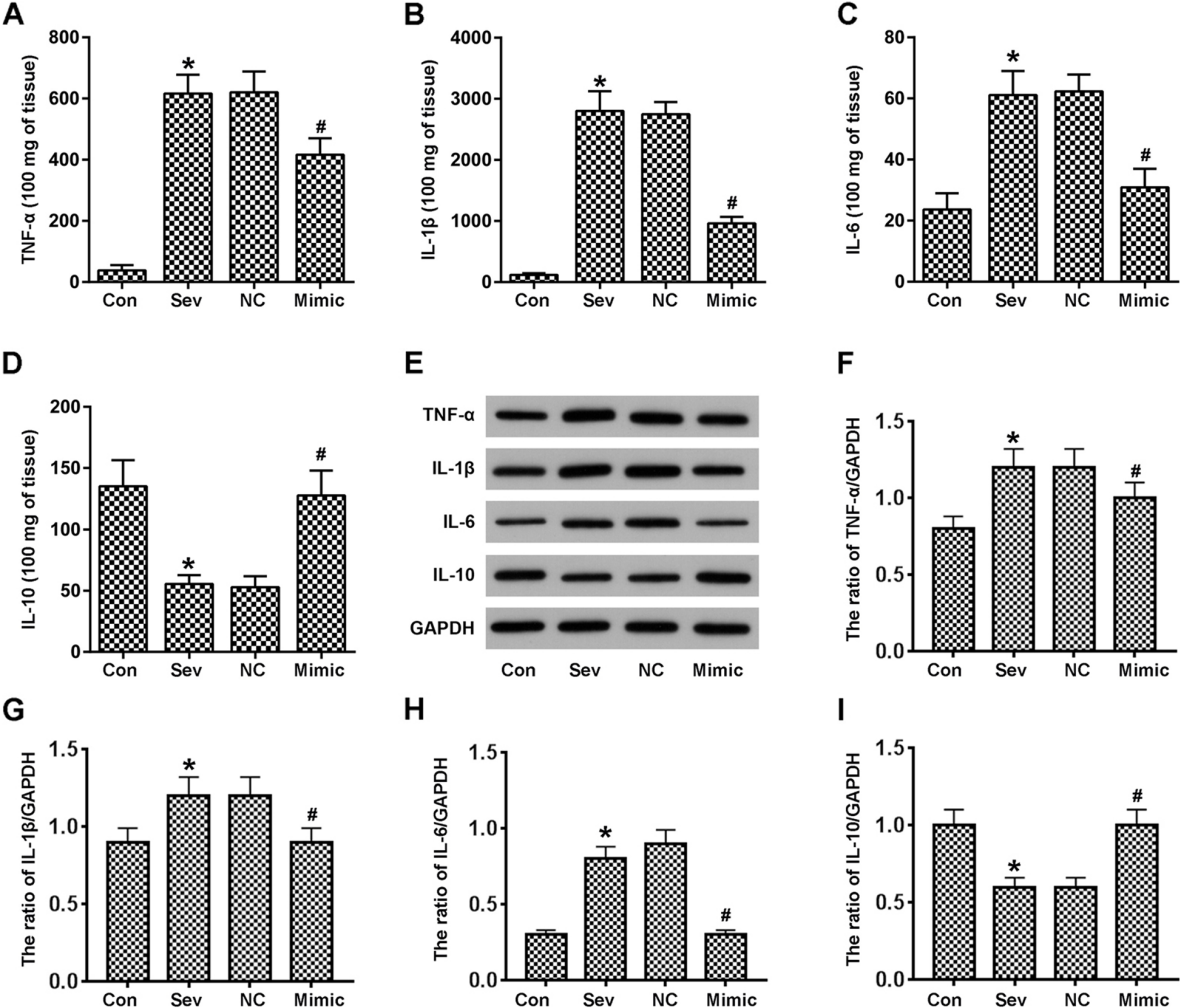
miR-424 inhibits hippocampal inflammatory responses in sevoflurane-treated rats. **A**–**D**. Inflammatory cytokines in vivo were detected by ELISA. **E**. Western blots were prepared for identification of inflammatory factors. **F**–**I**. Quantification by ImageJ software. n = 8; *p < 0.05 vs control; ^#^*p* < 0.05 vs NC

To further study the mechanism of sevoflurane in the hippocampus of rats, we conducted further experiments to assess inflammatory factors quantified by ELISA assay in different experimental groups. Sevoflurane strongly upregulated protein levels of TNF-α, IL-1β and IL-6, which was alleviated to some extent by exogenous treatment with miR-424 (Fig. 2A-C). In contrast, the expression of inflammatory inhibitor IL-10 in sevoflurane-treated groups was inhibited, while miR-424 reversed this effect (Fig. 2D). Western blot quantification was then used to verify these results (Fig. 2E-I). Compared with the control group, sevoflurane enhanced IL-1β, IL-6 and TNF-α protein expression and inhibited the expression of IL-10, while miR-424 weakened these effects.

### miR-424 inhibits TLR4/MyD88/NF-κB signaling pathway in vivo

**Fig. 3:**
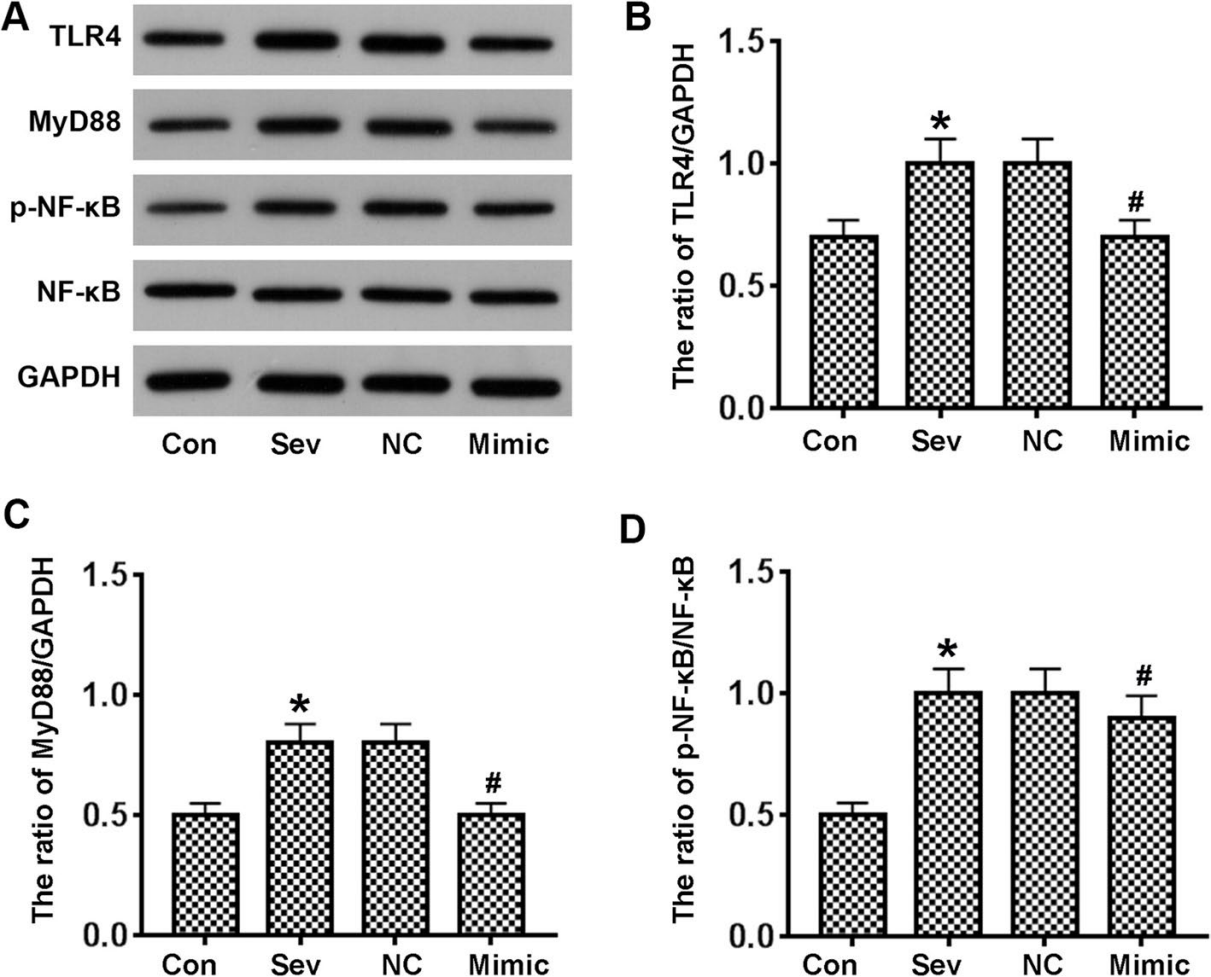
miR-424 inhibits TLR4/MyD88/NF-κB signaling pathway in the hippocampi of sevoflurane-treated rats. **A**. TLR4 regulatory factors in vivo were identified by western blot. **B**–**D**. Quantification of protein staining in 3A. *n* = 4; *p < 0.05 vs control; ^#^*p* < 0.05 vs NC

We next investigated the underlying mechanism for the inhibitory effect of miR-424. Through sequence alignment, we found that miR-424 and the 3′-UTR region of TLR4 shared a completely complementary sequence that was six bases long. The TLR4, MyD88 and NF-κB signaling factors were then detected by western blotting and subsequently quantified (Fig. 3). Our results indicated that TLR4 and MyD88 were significantly up-regulated and that phosphorylation of NFκB was significantly enhanced after sevoflurane treatment. On the other hand, we found that miR-424 inhibits this endogenous effect. To reveal the mechanism of this reaction, we designed in vitro molecular experiments.

### miR-424 interferes with the TLR4/MyD88/NF-κB pathway by targeting the 3′-UTR of TLR4 in vitro

**Fig. 4:**
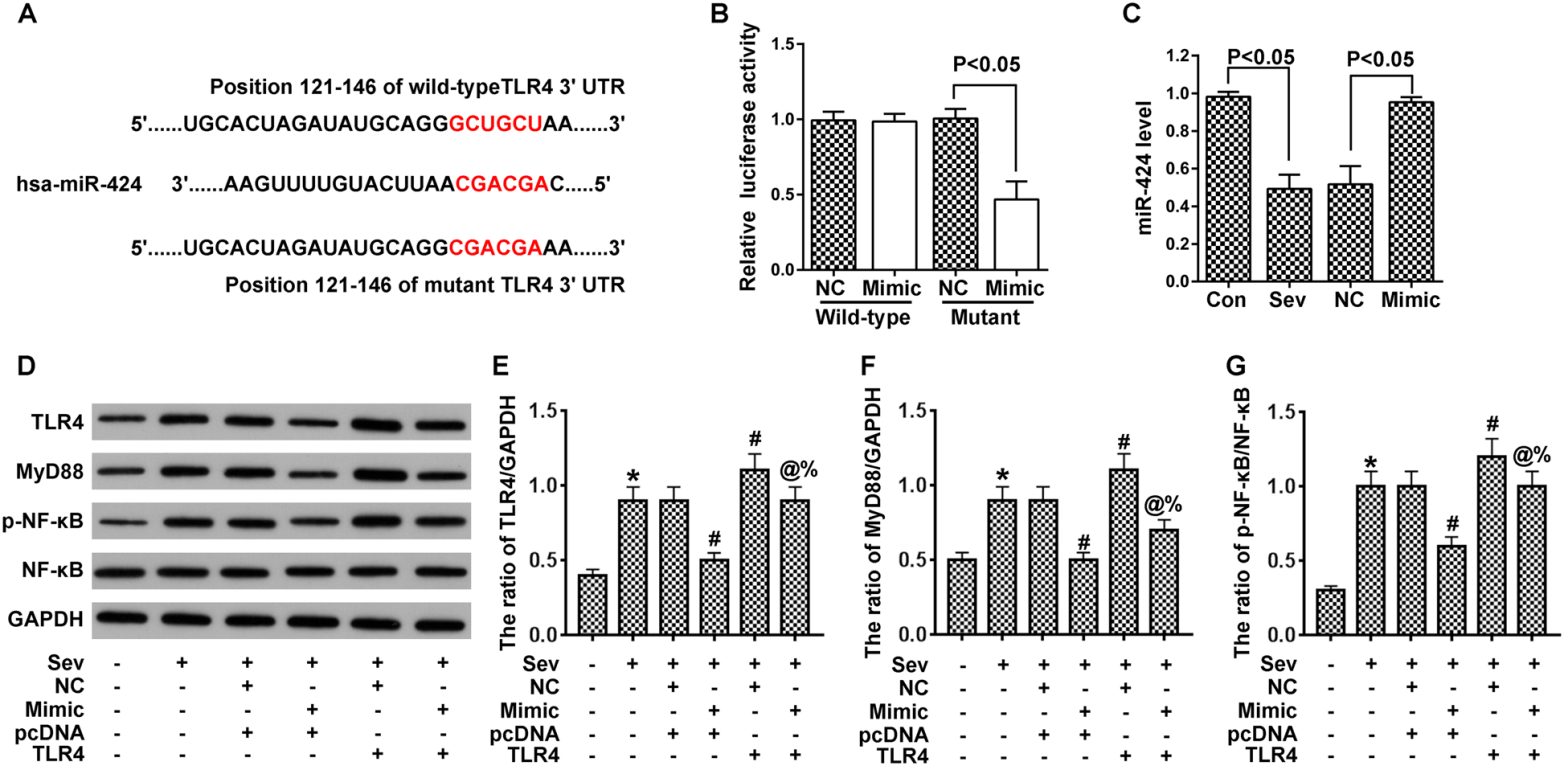
TLR4 is a direct target of miR-424 in PC12 cells. **A**. Diagram of complementary pairing between miR-424 and wild-type TLR4 or mutant TLR4 3′-UTR. **B**. Interaction between miR-424 and wild-type or mutant TLR4 was detected by dual luciferase report assays. **C**. Validation of miR-424 expression in PC12 cells with RT-PCR. **D**–**G**. Western blot was used to detect TLR4/MyD88/NF-κB signaling pathway-associated proteins. Images were quantified by ImageJ software, and the ratios of TLR4/GAPDH, MyD88/GAPDH, and p-NF-kB/NF-kB were calculated. *p < 0.05 vs control; ^#^*p* < 0.05 vs Sev + NC + pcDNA; ^@^*p* < 0.05 vs Sev + Mimic+pcDNA; ^%^*p* < 0.05 vs Sev + NC + TLR4

Complementary pairing between the TLR4 3′-UTR and miR-424 led us to design plasmids containing wild-type or mutant 3′-UTR sequence (Fig. 4A). Through luciferase report assays, we found that compared with NC miRNA, miR-424 significantly inhibited the expression of mutant TLR4, whereas miR-424 did not affect wild-type TLR4 (Fig. 4B). The expression level of miR-424 in different experimental groups was then checked through RT-PCR. Sevoflurane reduced transcription of miR-424 compared to the control group, and miR-424 mimic reduced this inhibition (Fig. 4C). We found that sevoflurane increased the expression of TLR4 signaling pathway factors in vivo, while miR-424 counteracted this effect. On the other hand, exogenous TLR4 also activated the TLR4-MyD88-NFκB signaling pathway, and this effect was stronger than sevoflurane treatment. Finally, all of these effects could be inhibited by miR-424 (Fig. 4D-G).

### Exogenous TLR4 counteracts the inhibitory effect of miR-424 on PC12 cells’ apoptosis

**Fig. 5:**
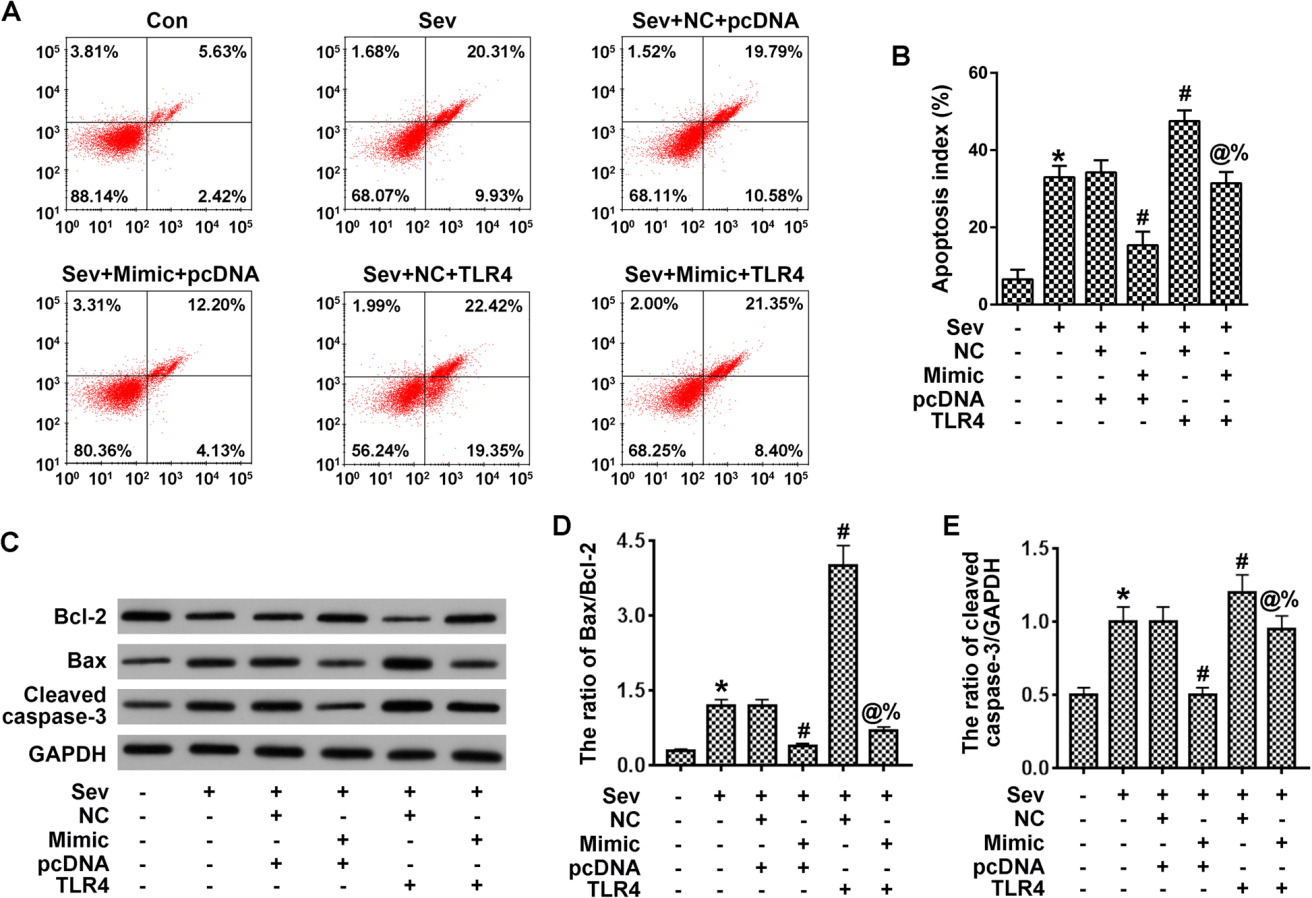
Overexpression of TLR4 inhibits the effects of miR-424 on apoptosis in sevoflurane-treated PC12 cells. **A**-**B**. Cell apoptosis was detected by flow cytometry followed by quantification. **C**–**E**. Western blots were used to detect key factors of apoptosis in vitro, followed by quantification. *p < 0.05 vs control; ^#^*p* < 0.05 vs Sev + NC + pcDNA; ^@^*p* < 0.05 vs Sev + Mimic+pcDNA0; ^%^*p* < 0.05 vs Sev + NC + TLR4

We carried out in vitro experiments in the PC12 cell line, and detected apoptosis via flow cytometry. Compared with the control group, sevoflurane treatment promoted apoptosis, and so did TLR4 overexpression, while miR-424 mitigated these effects. Our study indicated that TLR4 overexpression could counteract the inhibitory effect of miR-424 on apoptosis. miR-424 had a positive anti-apoptotic effect, and this effect resulted from its inhibition of TLR4 expression, which indicated that miR-424 could not continue to function after depletion (Fig. 5A, B).

We further examined the expression levels of apoptotic factors in vitro and found that sevoflurane downregulated Bcl-2 and upregulated the level of apoptotic factors Bax and caspase-3. Additional treatments also showed that miR-424 inhibited the effects of sevoflurane. Overexpression of TLR4 produced a similar effect to that of sevoflurane, i.e., activating cells to enter the apoptotic process, whereas miR-424 reduced apoptosis by inhibiting the TLR4 pathway (Fig. 5C-E).

### Exogenous TLR4 inhibits inflammatory responses induced by miR-424 in vitro

**Fig. 6:**
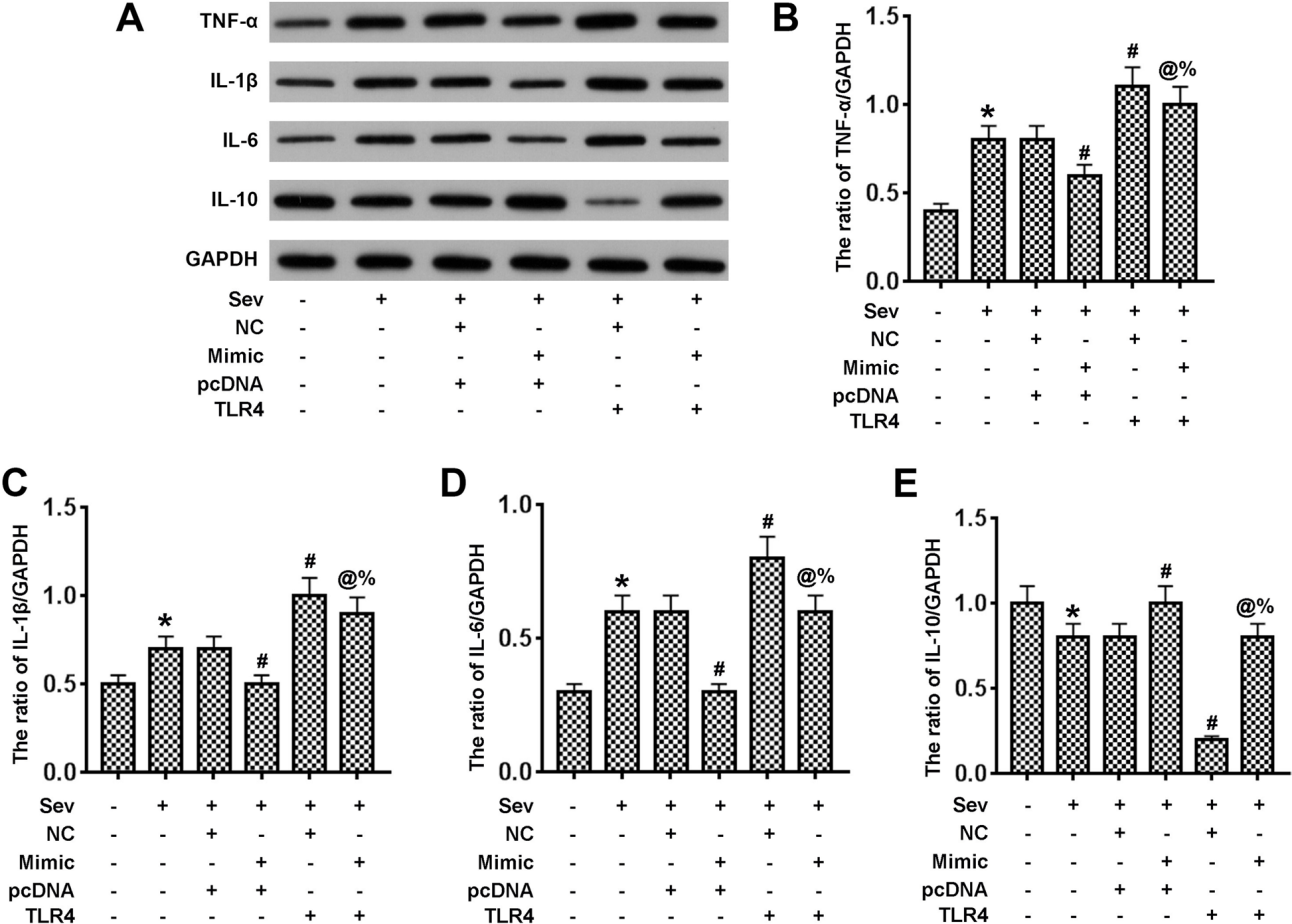
Overexpression of TLR4 inhibits the effects of miR-424 on inflammatory responses in sevoflurane-treated PC12 cells. **A**. Protein levels of inflammatory cytokines in different groups were monitored by Western blot and quantification. **B**–**E**. The ratios of TNF-α/GAPDH, IL-1β/GAPDH, IL-6/GAPDH, and IL-10/GAPDH. *p < 0.05 vs control; ^#^*p* < 0.05 vs Sev + NC + pcDNA; ^@^*p* < 0.05 vs Sev + Mimic+pcDNA0; ^%^*p* < 0.05 vs Sev + NC + TLR4.

Western blots were used to detect protein levels of inflammatory cytokines. In vitro, sevoflurane stimulated the expression of cytokines TNF-α, IL-1β and IL-6, while the expression of inflammatory inhibitor IL-10 was reduced. Similarly, TLR4 overexpression played a significant role in mediating inflammatory factors. Furthermore, miR-424 alleviated the pro-inflammatory effects of both sevoflurane and TLR4 (Fig. 6).

## Discussion

The anesthetic sevoflurane has many advantages, but its use also has limitations, including neuronal cytotoxicity [21, 22]. We examined the cytotoxic effect of sevoflurane on the hippocampus using rats as an in vivo model and PC12 cells for in vitro analysis. Our results showed that sevoflurane exposure was toxic to rat hippocampal neurons, and that apoptosis, inflammatory responses and immune induction were the main toxic effects [7].

TUNEL staining showed that apoptosis of hippocampal cells was increased after sevoflurane treatment, and the expression of apoptosis-related proteins was significantly up-regulated. The up-regulation of apoptotic factors Bax and caspase-3 suggested that sevoflurane induced oxidative stress leading to the progression of apoptosis [4, 5]. In addition, the up-regulation of pro-inflammatory markers was detected both by ELISA and western blotting, suggesting that the cytotoxic effect of sevoflurane also occurs through the activation of inflammatory signaling pathways. Our in vivo experiments showed that miR-424 inhibited the progression of apoptosis and inflammation in the hippocampus of rats, thereby improving outcomes.

The TLR4 signaling pathway is specifically related to the proliferation and differentiation of nerve cells [11, 13]. Our experiments showed that sevoflurane stimulated nerve cells apoptosis by promoting the activation of TLR4 signaling, which led to neurotoxic and side effects [13]. Western blot results showed that sevoflurane treatment significantly increased expression of TLR4, MyD88 and NF-kB, while miR-424 inhibited this effect. Thus, sevoflurane inhibits the differentiation and proliferation of nerve cells by promoting the activation of the TLR4 signaling pathway, resulting in neurotoxic side effects. In contrast, miR-424 inhibits translation of TLR4 by degrading transcripts after binding to the 3′-UTR region. The decrease in TLR4 protein levels stimulated the proliferation and differentiation of brain nervons which counteracted the negative effects of sevoflurane.

Both in vivo and in vitro experiments revealed that miR-424 inhibits TLR4 translation by targeting its transcripts, thereby inhibiting the TLR4/ Dym88 /NF-kB signaling pathway and improving clinical outcomes. In addition, miR-424 protects cell from apoptosis by inhibiting the expression of apoptotic factors and inflammation-related factors. That is, this study confirmed for the first time that miR-424 ameliorated the neurotoxic side effects of sevoflurane-induced anesthesia via regulating TLR4/ Dym88 /NF-kB pathway. However, issues such as improving the stability of miRNA and reducing its toxicity are still urgent problems that need to be solved before miR-424 can be applied in clinical treatment.

## Data Availability

The data used to support the findings of this study are available from the corresponding author upon request.
